# Recent advances in nanotheranostics for triple negative breast cancer treatment

**DOI:** 10.1186/s13046-019-1443-1

**Published:** 2019-10-28

**Authors:** Vikram Thakur, Rajaletchumy Veloo Kutty

**Affiliations:** 1 Department of Virology, Postgraduate Institute of Medical Education and Research, PGIMER, Chandigarh, 160012 India; 2 Faculty of Chemical and Process Engineering Technology, College of Engineering Technology,University Malaysia Pahang, Tun Razak Highway, 26300 Kuantan, Pahang Malaysia; 3 Center of Excellence for Advanced Research in Fluid Flow, University Malaysia Pahang, 26300, Kuantan, Pahang Malaysia

**Keywords:** Breast Cancer, Nanomedicine, Theranostics, Immunotherapy, NanoEL, Nanotechnology

## Abstract

Triple-negative breast cancer (TNBC) is the most complex and aggressive type of breast cancer encountered world widely in women. Absence of hormonal receptors on breast cancer cells necessitates the chemotherapy as the only treatment regime. High propensity to metastasize and relapse in addition to poor prognosis and survival motivated the oncologist, nano-medical scientist to develop novel and efficient nanotherapies to solve such a big TNBC challenge. Recently, the focus for enhanced availability, targeted cellular uptake with minimal toxicity is achieved by nano-carriers. These smart nano-carriers carrying all the necessary arsenals (drugs, tracking probe, and ligand) designed in such a way that specifically targets the TNBC cells at site. Articulating the targeted delivery system with multifunctional molecules for high specificity, tracking, diagnosis, and treatment emerged as theranostic approach. In this review, in addition to classical treatment modalities, recent advances in nanotheranostics for early and effective diagnostic and treatment is discussed. This review highlighted the recently FDA approved immunotherapy and all the ongoing clinical trials for TNBC, in addition to nanoparticle assisted immunotherapy. Futuristic but realistic advancements in artificial intelligence (AI) and machine learning not only improve early diagnosis but also assist clinicians for their workup in TNBC. The novel concept of Nanoparticles induced endothelial leakiness (NanoEL) as a way of tumor invasion is also discussed in addition to classical EPR effect. This review intends to provide basic insight and understanding of the novel nano-therapeutic modalities in TNBC diagnosis and treatment and to sensitize the readers for continue designing the novel nanomedicine. This is the first time that designing nanoparticles with stoichiometric definable number of antibodies per nanoparticle now represents the next level of precision by design in nanomedicine.

## Background

Breast cancer (BC) is the most common malignancy with 266,120 new cases and leading cause of cancer-related mortality (40,920 BC deaths) among women worldwide [1, 2]. Microarray-based expression profiling revealed the existence of five intrinsic subgroups of BC [3]. Triple-negative breast cancer (TNBC) owes 15-20% of all the invasive subtypes of breast cancer [4] and characterized by absence of expression of estrogen receptors (ER), progesterone receptors (PR) and human epidermal growth factor receptor 2 (HER-2) on tumors cell membrane. Based on the biological network-driven approach, *Bonsang-Kitzis et al* identified six TNBC subgroups whereas *Burstein at al* identified four stable TNBC subgroups based on mRNA expression and DNA genomic profiling [5, 6]. Lack of hormone receptors (ER/PR/HER-2) in TNBC eliminates the benefits of endocrine therapy and treatment, therefore mainly relies on chemotherapy [7]. Even systemic chemotherapy with clinically approved drugs reflects poor response, high toxicity and develops multidrug resistance. In addition, molecular heterogeneity, high risk to metastasize preferentially to the viscera, high relapse rate and *BRCA* mutations (BRCA*) contribute to poor prognosis and management [8–10].

For early therapeutic intervention, precise diagnosis is crucial. So far palpation, mammography, ultrasonography, ultrasound, magnetic resonance imaging (MRI) and immuno-histochemistry (IHC) are best TNBC diagnostics in the clinical setup. However, inaccurate diagnosis using non-specific contrast agents, false positive findings and examiner experience are the limiting and decisive factors to validate TNBC diagnosis. In addition, therapeutic interventions are limited to surgery, radiotherapy in addition to cytotoxic chemotherapy with taxanes and anthracyclines [11]. These limitations obviate the need to improve the currently available diagnostic and therapeutic in addition to explore the novel methods and approaches.

Last two decades of nano-technological advancements exploring biomedical science for cancer therapy with contrast agents and drug delivery carriers, now heading towards more precise and targeted co-delivery of both diagnostic and therapeutic agents. The availability of wide variety of nano-carriers were made from polymers, lipids, nucleic acid, proteins, carbon and metals including micelles, dendrimers, liposomes, nanoparticles/tubes, and DNA tetrahedral /pyramids [12–16]. These smart nanoparticles encapsulating anti-tumor drugs (*arsenal*), and surface coated with specific ligand (*key*) that eventually bind with the receptor (*lock*) expressing on the BC site (*target*) and destroy the cells in addition to molecular imaging (tracer agents) allowing us to simultaneously diagnose and treat the cancer i.e. *Thenanostic* approach for improving current cancer diagnosis and treatment regime. In recent years, theranostic approach has become more evident to develop efficient drug delivery system which will be able to cross the biological barriers for the delivery of right amount of drug at designated location and at/for appropriate time finally reduces side effects and improves therapeutic efficiency [17]. Although there is no FDA approved theranostic for TNBC, current approaches in conjugation with novel therapeutic modules are still indispensable need in clinical setup. As the therapeutic options for TNBC are limited, implementation of cancer immunotherapy has been successful in treating many malignancies. Recently, FDA granted approval to atezolizumab as first immunotherapy for TNBC treatment. So, it is worth exploring immunotherapies and performing clinical studies for treating TNBC patients with immunotherapy [18, 19].

### Triple-negative breast cancer: Current conventional diagnosis and therapeutics

In clinical setup, radiological, clinical, and pathological examinations are the main diagnostic approaches for BC diagnosis. Most widely applied radiological examination is mammography (using x-ray), but lack of abnormal features in TNBC tumors, resulting to an inaccurate diagnosis [20]. To overcome the mammography limitations, ultrasonography representing higher sensitivity (>90%) should be considered [21], but limited accuracy for benign tumors, restricts the use for TNBC detection. MRI is the sensitive with high positive predictive values in TNBC diagnosis, resulting in false positive findings which eventually lead to avoidable painful biopsies [22]. Accuracy of TNBC detection by above radiological examinations requires expertise and experience with clinicians to ever evolving radio-graphical technologies and new cancer/tumor modalities like benign or early stage cancer. So, the role of immunohistochemistry (IHC) and onco-pathologist/clinicians is crucial in the clinical identification of TNBC. The immunohistochemical identification of TNBC relies on the hallmark property of absence/lack of hormonal receptors (ER, PR) and HER-2 in patient’s biopsy tissues [23] and evaluated as best TNBC diagnosis.

After the proper TNBC diagnosis and considering other factors like metastatic nature, drug sensitivity/resistance, recurrence and poor prognosis, therapeutic intervention is done. Breast conservation treatment (BCT) is the first choice and attempt to avoid mastectomy in TNBC. However, the high incidence of tumor recurrence even after undergoing radiation treatment (RT), insist the patient for mastectomy in addition to radiotherapy [24]. Hormonal therapy which is successful in other subtype of breast cancer is not applicable to TNBC due to lack of HER2 and hormonal ER and PR receptors, thereby necessitates the chemotherapy, which is currently the mainstay of systemic treatment [25]. Chemotherapeutic drugs like anthracyclins and taxanes are commonly used for breast cancer treatment showing promising response in TNBC [26], but inherited cytotoxic effects and current non-targeted strategy of drug administration need to be resolved with novel technologies. Repeated chemo cycles with high doses of cytotoxic drugs destroy cancer cells in addition to the healthy cells in the vicinity. To avoid the non-specific targeting and chemo side-effect, nanotechnology based drug delivery systems are a promising tool. Recent advancements in nanotechnology and articulation of diagnostics with therapeutics in theranostic approach as a co-delivery system, not only the target the cancer selectively but also eliminate the cytotoxicity of drugs to other organ.

### Nanotechnology-advancements for TNBC: Targeted Theranostics

In nano-science, developing a promising nanoparticle entails numerous physiochemical, biological and functional properties for biomedicine application. The most important is the size; the desired size of nanoparticles (1-200 nm) and conformation decide the trajectory dynamics of the particles which is decisive for nanomedicine formulation. In addition, surface charge and encapsulation capacity of the nanoparticle are the key factors for the precise targeted drug delivery using specific conjugated ligand against the target receptor on cancer cell. Other properties like high drug loading efficiency, long half-life in circulation with minimum systemic toxicity, selective localization, high adhesion at the tumor environment, enhanced internalization into the tumor via endocytosis, sustained and controlled release of imaging agents and cytotoxic drug over right duration and time in addition to safe bio-elimination from the body are significant for nanoparticles to be as theranostics in cancer diagnosis and treatment [27]. Majority of the above nano-delivery systems rely on the enhanced permeation and retention (EPR) effect for targeted drug delivery. Technical feasibility (high recovery with controlled drug loading and releasing) and financial stability for the large-scale production, also determine the success and research on cancer nano-medicine. However, application of nano-medicine is limited in TNBC due to lack of known highly expressed tumor target and ligands.

#### Liposomal nanoparticles: A versatile spherical nanocarrier

Liposomes are spherical vesicles (400 nm) molecule with central aqueous core surrounded by lipid bilayers (Table 1). The feasibility to encapsulate drug either in lipid membrane or inside the aqueous core, mark the liposomes the most versatile nanocarriers with better drug distribution. Generally, liposomal nanoparticles are designed by the different methods i.e. extrusion (process of producing nanoparticles of fixed cross-sectional area), solvent injection (method of lipid precipitation from a dissolved lipid in solution) and reverse phase evaporation. *Dai et al,*[28] targeted the over-expressing integrin-α3 in TNBC models with cyclic octapeptide LXY (Cys-Asp-Gly-Phe (3,5-DiF)-Gly-Hyp-Asn-Cys) attached liposomes carrying dual drug i.e. doxorubicin and rapamycin (Table 1). This dual drug targeted approach resulted in improved efficacy as compared to free drug. Similarly, enhanced antitumor activity in TNBC xenograft mice model has shown with doxorubicin and sorafenib loaded liposomes [29]. However, currently marketed doxorubicin liposomal formulations are associated with cardiotoxicity, a novel micelle-encapsulated doxorubicin formulation (NK911) with improved tumour penetration and reduced *in vivo* toxicity is in trial [30]. Liposomal drug delivery system for co-delivery of antagomir-10b (anti-metastasis) and PTX (anti-cancer) was developed to delay 4T1 tumour growth and reduce the lung metastases of breast cancer [31]. A significant inhibition and reduction of 82% in the tumor growth was observed with PEG coated PTX nanocrystals targeting nude mice (MDA-MB-231/luc) and a lung tumour metastasis model [32]. Much higher i.e. 87% inhibition of breast tumor growth was reported in xenografted mice (MDA-MB-231 cells) by lipid-conjugated estrogenic (bioactive; 47.03%) NPs in combination with cisplatin [33]. So far, paclitaxel and irinotecan loaded liposomes known as EndoTAG-1 and MM-398 reached the way to clinical studies in TNBC patients [34].

**Table 1:**
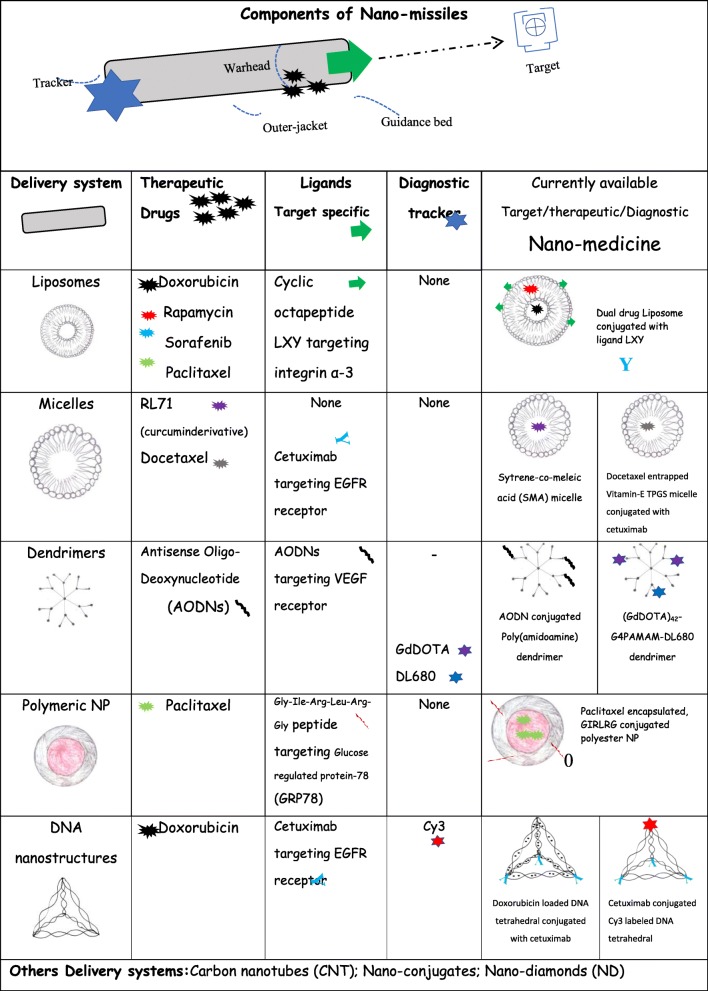
Targeted and drug delivery system: Liposomes, Micelles, Dendrimers, Polymeric NP and DNA nanostructure are the few delivery systems used to carry different therapeutic drugs like paclitaxel, doxorubicin and docetaxel in addition to tracking dye Cy3 as in DNA nanostructure for the precise and targeted delivery using the target specific ligand cetuximab

#### Micelles:A miracle ball in cancer therapy

**Fig. 1:**
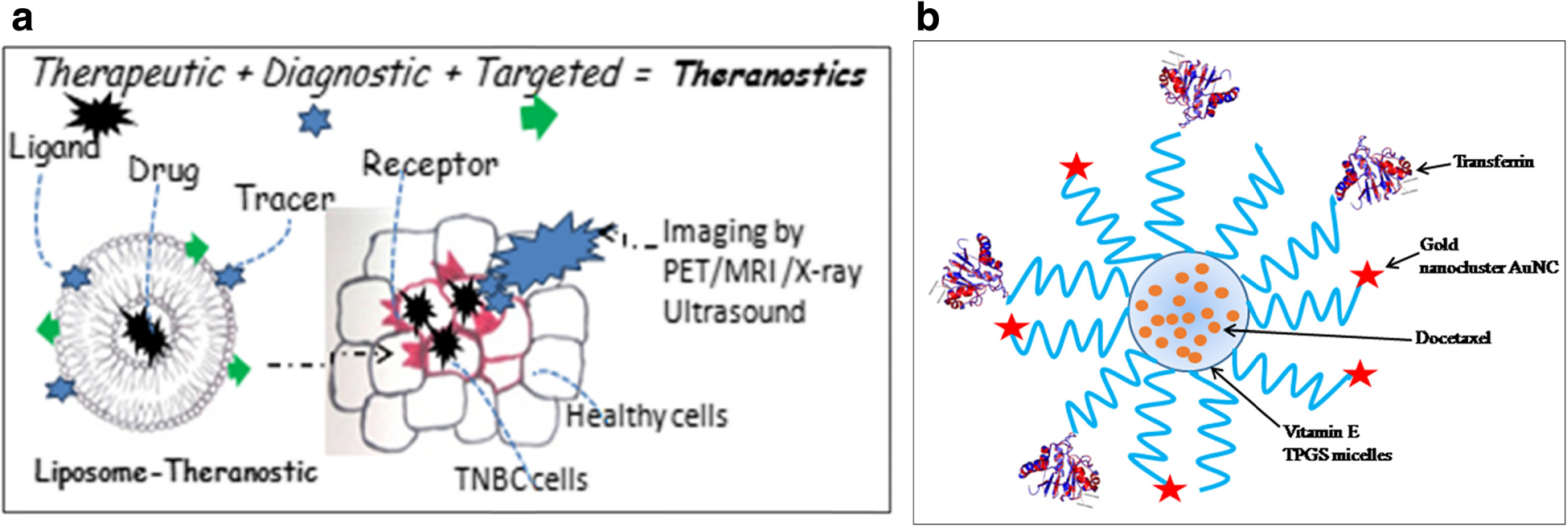
Diagrammatic representation of the concept of targeted theranostics: **a** Liposome cavity is loaded with the anti-cancerous drug and the surface of the liposomal nanoparticle is coated with ligand for the targeted and specific delivery of the drug. In addition to this, tracer helps in the accurate screening and diagnosis of cancer cells. **b** Transferrin is conjugated to vitamin-ED-alpha-tocopheryl polyethylene glycol succinate (TPGS) micelle for targeted co-delivery of therapeutic drug (docetaxel) and diagnostic agent (AuNC) as theranostic.

Micelles are colloidal particles (5-100 nm) with a hydrophobic core formed from Van-der Waals bonds and stabilized by hydrophilic shell [35]. Owing to its amphiphilic nature, micelle can deliver both water soluble and hydrophobic drugs for cancer therapy. *Taurin et al* [36] synthesized a micellar system using styrene-co-maleic acid (SMA) to deliver a hydrophobic curcumin derivative i.e. RL71 for TNBC treatment and showed higher toxicity to cancer cell due to endocytosis mediated higher cellular uptake and slow release profile (Table 1). Although, above strategy enhanced the drug uptake but it lacks specificity which is still a serious challenge in the treatment of metastatic TNBC. Utilizing the concept of specific ligand-receptor interaction and the fact that cetuximab (human chimeric monoclonal antibody) targets the epidermal growth factor receptor (EGFR) over-expressed in TNBCs, *Kutty and Feng et al* [12] developed cetuximab-conjugated micelles of vitamin E D-alpha-tocopheryl polyethylene glycol succinate for the targeted delivery of docetaxel drug (Fig. 1) (Table 1). *In-vitro* experiments in high EGFR expressing TNBC cell line (MDA MB 468), with different formulation of micelles showed the IC_50_ of 0.1715 μg/ml for TPGS micelle with cetuximab, in comparison to IC_50_ of 1.12 and 35.26 μg/ml respectively for TPGS micelle without cetuximab and free drug. These results have a promising utility in TNBC treatment subjected to further clinical trials and could be explored as theranostics. A classic example of theranostic and its application in cancer medicine is given by *Muthu et al* [37] where they developed TPGS micelle conjugated with ligand transferrin which mediated co-delivery of therapeutic docetaxel (drug) and diagnostic nanoclusterAuNc (imaging) for simultaneous detection and treatment in transferrin receptor expressing MDA-MB-231-Luc breast cancer *in vitro* model. Real-time imaging and tumor inhibition were imaged in xenograft model using above delivery system. Poly (acrylic acid)-g-PEG i.e. PAA-g-PEG copolymeric micelles carrying DOX (50 wt/wt %) was developed by *Sun et al* [31] for the efficient reduction in lung metastasis and 4T1 mouse breast tumor growth. However, the only miracle micelle that entered the phase-II clinical trials in TNBC patients is SN-38 (irinotecan) carrying poly(ethylene-glycol)-poly(glutamic-acid) PEG-PGlu i.e. NK012 micelle [38] and this needs to be validated in other phases of clinical trials.

#### Dendrimers: A nanovehicle for siRNA delivery

Dendrimers are synthetic macromolecules (10 nm to 100 nm) prepared by either divergent or convergent synthesis of branched monomer. Like liposomes, it presents a cavity-enriched spherical shape with a hydrophobic core and hydrophilic periphery, making them a unique vehicle for siRNA delivery [13, 39]. *Wang et al*.,[40] demonstrated reduction in tumor vascularization in TNBC xenograft mouse model using antisense oligo (AODNs) conjugated poly(amidoamine) dendrimers. This targeted therapeutic approach utilizes the higher expression of vascular endothelial growth factor (VEGF) as receptors of AODNs (Table 1). In another targeted therapy, utilizing siRNA conjugated poly(amidoamine) dendrimer, *Finlay et al.,* [41] have shown the down-regulation of a promising TNBC target i.e. TWISTItranscriptor factor. Dendrimer was also assessed as a targeted diagnostic module by *Zhang et al* [42] in TNBC tumor mice model. A novel dendrimer G4PAMAM conjugated with GdDOTA (MRI contrast) and DL680 (NIR dye) was prepared and injected subcutaneously in mice as a dual model for imaging and drug delivery (Table 1). MRI scan and near infra-red (NIR) fluorescence imaging showing homing of nanoparticles and higher fluorescence signal respectively in the TNBC tumor, demonstrated targeted diagnostic application of this small sized (GdDOTA)_42_-G4PAMAM-DL680 dendrimeric agent.

#### Polymeric nanoparticles: A misnomer nanoparticle for cancer treatment

Polymeric nanoparticle (50 nm-10 μm), if up to 10 microns in size; can be classified as misnomer nano-particle. With an additional advantage of encapsulating drugs and proteins without chemical modification, these nano-particles can be prepared from natural or synthetic polymer. Owing to the biocompatibility and reduced toxicity property, biodegradable polymeric particles viz. poly(lactic) and copolymer like poly(lactide-co-glycolide) are being in application for nanoparticle synthesis [43]. Drug molecule could be efficiently encapsulated by the method of nanoprecipitation, electrospray and emulsification; however, *Xu et al* [44] developed a novel approach known as PRINT for the synthesis of uniform sized polymeric nanoparticle. PRINT i.e. particle replication in nonwetting templates method provide room for properties customization for effective cancer therapy. Non-targeted delivery of Pt (IV) mitaplatin drug using PLGA-PEG i.e. poly-D, L-lactic-co-glycolic-acid – block-poly-ethylene-glycol nanoparticle, showed higher degree of tumor inhibition in *in-vivo *TNBC mice model (nude mice with MDA MB 468 TNBC cells) [45]. *Passarella et al.,*[46] identified a novel peptide (Gle-Ile-Arg-Leu-Arg-Gly) which specifically recognizes glucose-regulated protein (GRP78) (Table 1). Using irradiated TNBC xenograft mice model expressing GRP78 receptor; this group precisely reported apoptosis at the tumor site by target specific GIALAG-conjugated paclitaxel encapsulated polyester nanoparticles. In a recent clinical trial, 33% response rate was observed in 90% of the pre-treated metastatic TNBC patients expressing high protein Trop-2 with IMMU-1322 drug (anti-Trop-2-SN-38 antibody) [47]. Succinobucol with P188 (poloxamer) combination are emerging as a best oral treatment for breast cancer. Better bioavailability (13 fold) of succinobucol NPs enhances the inhibition of vascular cell adhesion molecule-1 (VCAM-1) invasion and tumor cell migration [48]. Polymeric NPs are also known to deliver miRNA and siRNA along with therapeutic drug to reduce the tumor volume and ultimately tumor growth. PLGA-b-PEG polymer NPs co-delivered both antisense-miR-21 and antisense-miR-10b with 0.15 mg/kg drug dose whereas siRNA (multidrug resistance protein) and DOX co-loaded NP caused overall reduction in tumor growth and volume (8-fold decrease) respectively [49, 50].

A promising ligand, Arg-Gly-Asp (RGD), either facilitates targeted delivery of drug or inhibits invasion of cancer differently in TNBC tumor models. For instances, cyclic RGD-functionalized solid lipid NP (RGD-SLN) shown to inhibit adhesion and invasion of alphavbeta 3 (αvβ-3) integrin receptor over-expressed in invasive TNBC tumors [51]. This is the perfect example of ligand targeting and inducing inhibition simultaneously in breast cancer cell. Similarly, *Zhang et al.,* [52] synthesized hybrid shealth polymer-lipid nanoparticles (PLN), conjugated to peptide ligand RGD and co-loaded with doxorubicin (DOX) and mitomycin C (MMC) i.e RGD-DMPLN. The targeted therapeutic efficiency of RGD-DMPLN was assessed in metastatic TNBC mouse model developed using MDA-MB-231-luc-D3H2LN cell line. Enhanced cytotoxicity in both above models was achieved by the virtue of DOX-MMC synergism which further enhances by the target RGD-DMPLN. This type of targeted delivery of synergistic drug enhances the overall efficacy in cancer treatment and need to explore more for wider application in breast cancer.

#### DNA nanostructures in cancer therapy: DNA beyond coding secrets of life

DNA nanostructures utilize the most fundamental property of DNA i.e. Watson-Crick complementary nucleic acid base pairing to design different nanostructure like tetrahedral, bipyramids, cages, and cubes with desired shapes, sizes and configuration. These DNA nanostructures can incorporate ligands and/or small functional compounds for site specific attachment and/or for bio-imaging. *Kutty et al.,* [27] designed a novel self-assembled DNA nanopyramid, tagged with red-emissive glutathione-protected gold nanoclusters (GSH-Au NCs) at the base and actinomycin (AMD) incorporated in the DNA minor groove. This theranostic DPAu/AMD so far developed for detection and killing of *Escherichia coli* and warrant evaluation and modification for other disease/cancer. One of the major challenges utilizing these structures is to escape the endosome degradation of DNA nanostructure in mammalian TNBC. However, same group developed another nanostructure, i.e. DNA tetrahedral (TH) for bio-sensing and antibody-mediated targeted drug delivery. DNA tetrahedron self-assembled to form four vertices. Cetuximab conjugated TH (THC_3_) with intercalated doxorubicin (DOX) drug i.e. THDC_3_ (Table 1) showed preferential killing of MDA-MB-468 cancer cells, due to cetuximab which is known to target EGFR over-expressing cancer cells. Low IC_50_ value of THDC_3_ i.e. 0.91 μM in comparison to free DOX i.e. 3.06 μM signifies the high and specific killing efficiency of THDC_3_ [16]. Another modified formulation carrying one Cy3 probe and three cetuximab i.e. Cy3-THC_3_ shows high signalling intensity due to increased uptake of Cy3-THC_3_ into MDA-MB-68 cells. These two (THDC_3_ and Cy3-THC_3_) slight modifications of TH show enhanced targeting and killing of cancer cells which could be an excellent candidate for cancer nano-medicine especially for TNBC.

#### Metal nanoparticles: Multifunctional smart hard materials for cancer therapy

In addition to above discussed nanoparticles, metallic NPs such as gold (Au), silver (Ag), platinum (Pt), zinc (ZnO), titanium dioxide (TiO_2_) and many others are used in cancer medicine. These nanoparticles may offer wide opportunity in therapeutic and diagnostic assay due to their magnetic, optical, thermal and electrical properties. Surface modification by conjugating different groups on metal NPs expands the utility for desired clinical outcomes. Different metal NPs utilizes diverse molecular mechanism like production of intracellular reactive oxygen species (ROS), increasing oxidative stress and specific apoptotic tumor cell death [53]. NPs from the transition class of metals induce hyperthermia (non-invasive method), to heat up the cells, thereby killing the tumor cells by converting electromagnetic radiations to heat. Few metal NPs have inherent potent anti-cancer activity owing to their unique physiochemical properties.

Gold nanoparticle (AuNPs) is the most extensively investigated and promising metal NP known to deliver paclitaxel, a widely known anti-cancer drug. Au NPs designed and synthesised in different shapes and configurations as Au-nanoshells (AuNS), Au-nanorods (AuNR) and Au-nanocages (AuNC) are now emerging as a versatile nanovehicle for cancer therapy. PEG coated Au NP in addition to ionizing radiations provided higher survival rate in mice model of breast cancer [54]. Serum-coated AuNR have inherited ability to down regulate the energy generation-related genes expression. Due to reduced energy, migration and invasion of cancer cell is inhibited in both *in-vitro* and *in-vivo*. *Andey et al.,*[33] also showed the inhibition/suppression of the TNBC tumor and metastasis using the combination of cisplatin loaded AuNR and NIR laser. Silver nanoparticles (Ag NPs) are known for its antiproliferative, proapoptotic, and anti-angiogenic effects on cancer cells. As a radiosensitzing agent, AuNPs reacts with acidic environment in cancer cells and increases oxidative stress by the production of ROS which eventually induce damage and apoptosis. *Liu et al*.,[55] observed promising results of AgNPs treatment followed by radiotherapy on gliomas. These NPs also observed to inhibit endothelial growth factor (VEGF) on cancer cells thus limiting the metastasis. Zinc oxide nanoparticles (ZnO NPs) function like genotoxic drugs for cancer treatment. ZnO NPs form micronucleus inside the tumor cell, which finally increase mitotic and interphase apoptosis death of the cell [56]. Asparaginase is a well-known anticancerous enzyme used as a chemotherapeutic agent in other cancer treatment, so ZnO NPs carrying asparaginase, further increase the specificity and stability when given in combination with paclitaxel and daunorubicin [57]. Even, ZnO NPs in combination with drugs paclitaxel and cisplatin shows reduced toxicity and increase efficacy in breast cancer cells [58].

Other metal NPs viz. copper (CuO NP), iron-oxide (Fe_2_O_3_), silica, cerium oxide and titanium oxide are also being explored and used in breast cancer diagnosis and treatment. Copper oxide NPs (CuO NPs) are described as green NPs as they were synthesized from *Ficus religioss* and *Acalypha indica*. Metastatic lung tumors of mouse (B16-F10 cells) are treated using CuO NPs by the mechanism of apoptosis and ROS generation [59]. Dual modal therapy employing photodermal and radiotherapy with Cu-64 labelled copper sulfide NP (CuS NP) showed the suppression of tumor growth in subcutaneous BT474 breast cancer model and prolonged the survival of mice bearing orthotopic 4T1 breast tumors [60]. Human breast carcinoma cells (in-vitro) and HER2+ breast carcinoma cells are specifically targeted by anti-HER2 antibody conjugated silica-gold nanoshells in photothermal therapy. Cerium oxide NPs (CNPs) function as a radiosensitizing agent thereby increases the oxidative stress and apoptotic tumor cell death by following the biological mechanism of DNA damage [61]. CNPs also supplement the conventional chemotherapy by delivering chemotherapeutic drugs like DOX, which provide a smart approach for cancer therapy. In addition, platinum and titanium-based NPs are also perceived as a promising nano-carriers and therapeutic candidate in cancer photodynamic therapy respectively. An iron-oxide nanoparticle (Fe_2_O_3_ NPs) explores the magnetic property for accurate diagnoses and targeted treatment of cancer as in squamous cell carcinoma mouse model [62]. A multivalent pseudopeptide (N6L) and doxorubicin (DOX) conjugated to Fe_2_O_3_ NPs (MF66) forming a multifunctionalised Fe_2_O_3_ NPs known as MF66-N6L-DOX. This system by combining both hyperthermia and drug delivery module, presented a better specificity and tumor killing potential in breast cancer model (athymic nude mice )[63]. Diagnosis of micrometastasis (0.5 mm dia) and metastatic breast cancer in transgenic mouse model are improved by cRGD conjugated Fe2O3 NPs and anti-neu receptor MAb conjugated superparamagnetic iron oxide NPs (SPIONs) respectively [64, 65]. Finally, trastuzumab conjugated modified magnetic polymerosomes named as herceptin is in clinical trials which can target bone metastasis in a HER2+ breast cancer model (BT474) of NOD/SCID mice.

#### Carbon nanotubes (CNTs): Folded grapheme for cancer therapy

Carbon nanotubes (CNTs) are benzene ring knitted flat sheets, folded to form single and/or multi-walled cylindrical structures. Slight chemical modification imparts multiple functions with the huge possibility in cancer therapy. Single walled NTs (1 nm-2 nm diameter) having the ability to penetrate inside cells shows prolonged distribution and localized effects. Oxidized multi-walled carbon nanotubes (o-MWNTs) present a novel approach in cancer therapy, by reducing macrophages and vessel density in the tumor [35, 66]. *Burke et al.,* [67] conceptualize the fact that by hyperthermia, NT promotes cell membrane permeabilization resulting in tumor mass destruction. So, he proposed photo-thermal-induced ablation using multi-walled NT for TNBC therapy. A complex of nanodiamond and DOX known to inhibit cancer in mouse model by overcoming drug efflux and increasing apoptosis [68] and lung metastasis of breast cancer [69].

### Ligands for targeted TNBC therapy

**Fig. 2:**
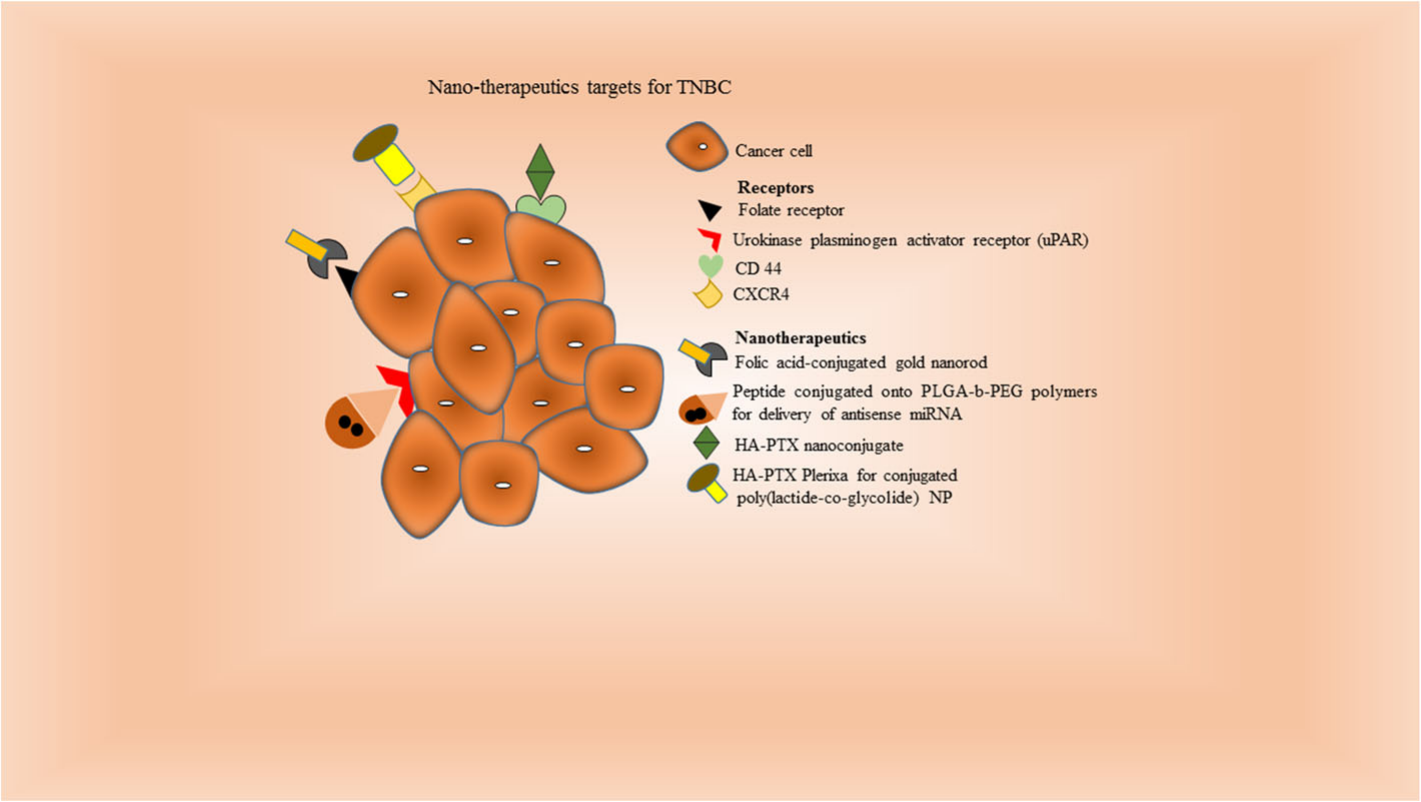
Diagrammatic representation of ligands and their specific receptors: Folate receptor is targeted by folic acid-conjugated gold nanorod as nanotherapeutics. Similarly, various other receptors like CD44 and CXCR4 are identified by HA-PTX nanoconjugate and HA-PTX poly(lactide-co-glycolide) nanoparticle respectively increases the specificity and decrease the adverse effects of the cancer therapy.

Ligands are the small stretch of nucleotides, peptide or small molecules itself which bind specifically to its receptor by ligand-receptor interaction. Few of the ligands were already discussed in the section 3. However other like aptamers, antibodies, peptides and other small molecules like carbon and quantum dots are also widely known ligands usedfor targeted or probe based diagnostic in cancer nanomedicine (Fig. 2).

#### Aptamers: Nucleic acid-based ligands

Aptamers are short oligonucleotides stretches of single-stranded DNA/RNA. Owing to unique 3D confirmation of aptamer, it specifically binds the target molecule with high affinity and strength. The only limitation is the degradation by nucleases; however, its high stability gained attention for the development of molecular probes. *Li et al.,*[70] in their preliminary study, specifically target a surface membrane protein on TNBC tumor by newly identified LXL-1 aptamer using cell-SELEX method. Differential overexpression of platelet derived growth factor (PDGF) receptor in TNBC cell line was detected by *Huang et al.,*[71] using PDGF-aptamer conjugated to gold nanoparticles. MCF7 and MDA-MB-415 breast cancer cells known to over express the mammaglobin A2 and mammaglobin B1. *Hassann et al*.,[72] detected the metastatic breast cancer by MAMA2 and MAMB1 aptamers using highly sensitive terahertz (THz) chemical microscopy (TCM) using THz radiations. Another 26-mer G-rich DNA aptamer specifically target the nucleolin receptor in some breast cancer cells [73]. However, such aptamer based precise targeted diagnosis still need to be improved and combined with drug delivery for TNBC theranostic application.

#### Antibodies: Y shaped key with dual functionality

Antibodies are Y-shaped protein with two epitopes, which has high selectivity and affinity for its receptor. These are rated as the best class of targeting ligands. The utility of antibodies in cancer diagnosis surpasses its high production costs. Conceptualizing the differential up-regulated expression of tissue factor (TF) receptor and urokinase plasminogen activator receptor (uPAR) in TNBC, *Shi et al*., [74] suggested and validated the application of the anti-TF antibody labeled with copper-64 (anti-TF-antibody-^64^Cu) using PET imaging in *in-vitro* TNBC model. *Le Beau et al*.,[75] detected NIR fluorophore and Indium-111 (^111^In) labelled uPAR antibodies using optical and SPECT imaging respectively. Similarly, anti-EGFR and anti-VEGFR antibodies conjugated with fluorescent NP and ultrasound contrast agents are detected using fluorescence microscopy and ultrasonography. Preclinical study on TNBC xenograft mice by *Rousseau et al.,* [76] demonstrates good visualization of TNBC tumor with Iodine-124 (^124^I) labeled B-B4 antibody (targeting syndecan-1; CD138 antigen) and experience good response (treatment) with I-131 (^131^I) radiolabelled B-B4 antibody.

#### Peptides: Cell penetrating ligands as diagnostic/imaging sequences

Peptides are low molecular weight ligands with an ability to target intracellular molecules with high specificity [77]. These target binding peptides sequences can fuse to bacterial coat proteins and expressed using genetic engineering which are finally screened by phage display library technique [78]. Few peptides for targeting metastatic breast cancer are RGD, P-selection, tumor metastasis targeting (TMT), and chlorotoxin. *Feng et al.,* [79] observed binding of CK3 peptide (Cys-Leu-Lys-Ala-asp-Lys-Ala-Lys-Cys) to NRP-1 trans-membrane protein (neuropilin-1) by NIR fluorescence imaging in breast cancer of TNBC mice models. Activable cell-penetrating peptide (ACPPs) which targets the matrix metalloproteinase (MMP)-2 enzymes, when covalently linked to cyclic-RGD peptide, resulted in enhanced tumor uptake and contrast imaging in *in-vivo* TNBC models [80]. Modified Fe_2_O_3_ NPs linked to cyclic RGD peptide resulted in superior and efficient targeting of αvβ3 integrin receptors [81]. Even the dual ligand (P-selectin and RGD-peptide) linked liposomal NP can capture different tumour sites over expressing their respective receptors on the breast cancer cells [82]. Difference in pH exploited using pH low insertion peptide (pHLIP). *Ali et al.,*[83] designed a pH-responsive MRI nano-probe i.e. pHLIP-conjugated MRI-NP which specifically internalize and accumulate in *in-vitro* TNBC cells in response to its low pH.

#### Other small molecules

These ligands (<500 Da) are the potential targeting agent for cancer imaging. The most widely clinically accepted molecule is ^18^F-FDG which is a glucose analogue [84] while other molecule like folate has potential as direct imaging agents. *Meier et al.,* [85] shows that folate molecule drives super-paramagnetic iron oxide contrast agent (P1133) to folate receptors and internalized in the actively growing TNBC in both *in-vitro* and *in-vivo* system. Even folic acid conjugated AuNR target the folate receptor and showed enhanced uptake in 4T1 metastatic breast cancer cells [86]. Carbon dots (CDots) and quantum dots (QDs) are useful in biomedical imaging [87] and holds great promise for early stage TNBC detection. Chemokine receptor type 4 (CXCR4) is a cellular target involved in the growth and metastasis of TNBC. Plerixafor or AMD3100 (CXCR4 ligand) conjugated poly(lactide-co-glycolide) NPs enhanced siRNA-mediated gene silencing by improving the cellular uptake into MDA-MB-231 cells [88]. Similarly, AMD3100 loaded human serum albumin encapsulated NPs targets CXCR4 on lung metastatic model of breast cancer [89]. Hyaluronic acid (HA) has high affinity to CD44 receptor therefore an ultra-small (~5 kDa) HA-PTX nanoconjugate are taken up via CD44 receptor-mediated endocytosis into metastatic breast cancer (MDA-MB-231Br) cells [90]. Urokinase plasminogen activator receptor (uPAR) targeting peptide conjugated to poly (lactic-co-glycolic acid)-b-PEG polymers carrying two antisense miRNA showed significantly higher tumor inhibition using [49]. Functionalized fullerenes have been used as novel contrast agents in MRI. Other small carbon molecules like nanocarbons, nanodiamonds with distinctive physical and chemical properties are also emerging in biomedicine [91, 92] and needs to be extensively studied.

### Virus like particles (VLPs) as novel nano-vehicles and future theranostics

Virus-like particles (VLPs) are self-assembled multimeric nanostructure (0.1- 100 nm) produced by the expression of viral structural genes in heterologous systems. The notion of *virus-like* to VLP is because they are free of any viral genetic material; and this makes them a versatile nanovehicle for drug delivery. VLP can be from microbial, plant or mammalian virus origin and assembled into spherical and filamentous [93]. Modified VLPs with foreign ligands is produced by expressing required heterologous peptides/proteins/gene sequence on the surface (capsomers). Also, chemical modification of the functional groups contained in the structural capsid protein aids targeted mediated therapy. Most remarkable attribute of VLPs are its small size enough to move in the blood stream and functional viral proteins on cell surface which facilitates cell entry/penetration inside the cell. The ability of VLP to encapsulate small molecules/drug may be applicable for cancer treatment by targeting and entering the specific tumor cells by energy-using receptor-mediated endocytosis and finally, liberating the encapsulated drug inside the cancer cell. Most astonishing ability is to escape the endosomes before lysosomal degradation; this favors the drug availability and protect drug in blood plasma. The only limitation with the use of VLP as drug delivery system is that it elicits innate immune response due to viral proteinaceous particle and readily up taken by dendritic cells [94], however on failure of classical chemotherapy, it gave an optimistic hope for TNBC treatment. Also increase in drug bioavailability and biocompatibility may compensate the above disadvantages**.** Various VLPs are derived from *Human papiloma virus* (HPV), *Bacteriophage*, *Polyomavirus*, *Ebola*, *Influenza*, *Hepatitis E virus* (HEV) [95] and *Tobacco mosaic virus* (TMV). Some VLPs display natural tropism to certain organ or tissues like HEV VLPs for liver/hepatocytes, however majority of the VLPs display tropism to sialic acids or heparin sulphates limits its use as a targeted nanocarrier. A classic example of VLPs as targeted therapeutic carrier is self-assembled *Bacteriphage MS2* VLP, which is modified with SP94 peptide and encapsulated with doxorubicin/cisplatin/ and 5-fluoro-uracil to selectively deliver and kill human hepatocellular carcinoma (HCC) in Hep3B cell line [96]. *Rotavirus shows* natural tropism towards the gut. These concepts utilize by *Cortes et al*.,[97] to develop *rotavirus* VLP which successfully enter (*in-vivo*) and deliver green fluorescent protein (GFP) in intestinal cells of healthy mice. Adenovirus (Ad3) derived VLP, dodecahedron chemically conjugated with anticancer antibiotic Bleomycin (BLM), Db-BLM induce death of transformed cells by causing ds-DNA breaks with lower concentration [98].

So, the popularity of VLPs is attributed by its versatility, cell-specific targeting, and efficient cell entry, lack of endosomal sequestering, multivalency, biocompatibility, large encapsulation and safe delivery system. Despite so many advantages, VLPs as drug delivery system are in their infancy and need to be validated on animal model.

### Need of nanomedicine for breast cancer therapy: Shift from conventional to nanomedicine

Conventional chemotherapeutic agents unfortunately associated with many limitations. Non-specific target resulting in systemic toxic effects, adverse clinical outcomes, toxic to rapidly dividing normal cells leading to chronic toxicity including very common manifestation like alopecia, mucositis and thrombocytopenia. Poor solubility and low bioavailability in addition to drug resistance due to possible mechanism involving overexpression of P-glycoprotein and mutated topoisomerase II further restricts the usefulness of anticancer agent. Even the tumor/cancer cells structural makeup limits the clinical outcomes due to poor penetration of drug because of physical barriers, intercellular junctions controlling drug permeation, and extracellular matrix proteins [99]. Current problem in cancer therapy is the rapid drug clearance and limited targeting, which necessitates the emergence of nanomedicine in treating cancer. Breast cancer primarily metastasizes to the regional lymph nodes, bone and lungs; however, metastatic breast cancer has spread to distant sites. Aggressive proliferation, heterogeneity and resistance of tumor to therapeutics are few challenges in treatment of metastatic breast cancer. Adjuvant therapy including chemotherapy (paclitaxel, eribulin), hormone therapy (letrozole, tamoxifen) has various long-term side effects affecting the patient’s quality of life [100]. So far, no targeted therapy in clinic for treating triple negative, resistant and recurrent breast cancer. Moreover, TNBC lacks ER, PR and Her-2 /neu and are also difficult to treat, therefore most likely to recur and disseminate. With characteristic short overall survival and increase risk of metastasis, its treatment remains a challenge. So, chemotherapy remains the only option for TNBC treatment with anthracyclin and taxane based chemotherapy and neoadjunant chemotherapy option [6, 101]. Despite comprehensive and aggressive management, 50% reoccurrence with 37% mortality necessitate the advanced, novel and effective therapy [102]. Therefore, multifunctional smart nanoparticles conjugated with targeting, therapeutic, fluorophore can cross different biological barriers, target and penetrating cancer cells by passive method known as enhanced permeability and retention (EPR) effect, and finally release drug in the cancer cells in a controlled manner.

#### Novel breast cancer drugs in useor in clinical trials

**Fig. 3:**
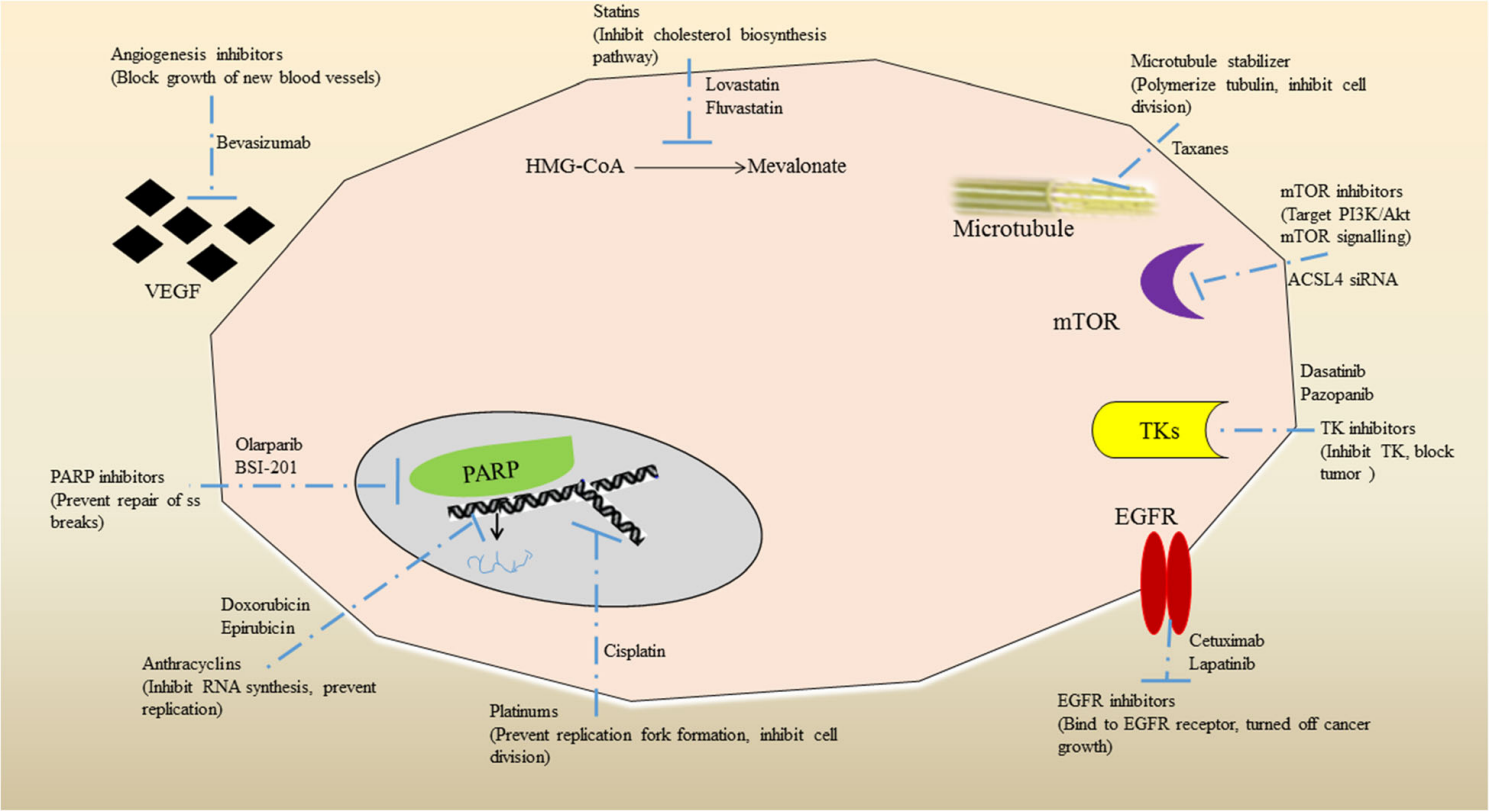
Brief representation of potential inhibitors of various pathways and receptors for the treatment of triple-negative breast cancer. Poly (ADP-ribose) polymerase (PARP) inhibitors like BSI-20I target ssDNA break repairing enzyme causing synthetic lethality resulting in control of cancer cell proliferation. Similarly, other class of inhibitors like tyrosine kinase (TK), EGFR, PI3K, angiogenesis, insulin-like growth factor (IGF), heat shock protein (Hsp90), histone deacetylase (HDAC), andmammalian target of rapamycin (mTOR) employ different mechanism to control and treat TNBC.

For the treatment of TNBC, many potential agents are under different stages of research and development. These potential agents/inhibitors have different specific targets and execute its anti-tumor activity differently [103]. A brief summary on different classes of inhibitors like poly (ADP-ribose) polymerase (PARP), tyrosine kinase (TK), EGFR, PI3K, angiogenesis, insulin-like growth factor (IGF), heat shock protein (Hsp90) and histone deacetylase (HDAC), mammalian target of rapamycin (mTOR), and the mechanism of their action shown in Fig. 3. Briefly, Poly (ADP-ribose) polymerase inhibitors (PARPI) target ssDNA break repairing enzyme causing synthetic lethality [104]. Various PARPIs like olaparib, veliparib, talazoparib have been evaluated in clinical trials on TNBC patients. Olaparib for BRCA-mTNBC is undergoing phase III (OLYMPIAD; NCT02032823) trial will likely to be completed in March 2020. Olaparib in combination with paclitaxel, cisplatin induced overall response rate of 88% [105]. Receptor tyrosine kinase (RTK) inhibitors targets in TNBC are EGFR, FGFR, VEGFR and MET. EGFR expressed in 89% of TNBC and appears a promising therapeutic target, but surprisingly majority of the EGFR-TKIs trials against TNBC are not promising [106]. FGFR as a therapeutic target in only ~10% TNBC emerged recently, therefore pan-FGFR inhibitors PD173074 and alofanib inhibits the proliferation of SUM52PE and induce apoptosis by inhibiting MAPK and PI3K signalling cascades [107]. VEGF expression are associated with poor prognosis in TNBC, however the clinical trials with bevacizumab and apatinib targeting VEGF2 do not produce promising results [108]. Contrary to this, sunitinib, anti-VEGFR tyrosine kinase inhibitors are emerging as a potential therapeutic candidate in breast cancer trials. MET is a TNBC cell surface RTK which activates multiple downstream effectors including Src, AKT, ERK and RAS. Phase II trial of the tivntinib (MET inhibitor) is disappointing, however MET+EGFR inhibition synergistically reduced cell viability, highlighting the superior efficacy of this combination [109].

Non-receptor tyrosine kinases (NRTKs) are cytoplasmic kinases including PI3-AKT-mTOR signalling cascade, Src, and MEK. Dual mTORC1/2 inhibitors with everolimus, synergistically reduce proliferation of multiple TNBC cell lines. However clinical trials on TNBC with mTOR+PARP inhibitors and dual mTOR/P13K inhibitors are ongoing. PI3-AKT-mTOR pathway represents as an emerging multi-target of drugs at early stages of clinical development [110]. MEK is a component of the MAPK signalling cascade where MEK inhibitors (U0126) significantly reduce the invasiveness of MDA-MB-2311 *in-vitro* whilst lung metastasis is known to be inhibited in xenograft model by selumetinib [111]. Src is a cytoplasmin oncoprotein and addition of Src inhibitors i.e. dasatinib to cetuximab + cisplatin enhanced the inhibition of cell growth and invasion in TNBC [112]. Epigenetic targets like HDACs and Hsp 90 are also being investigated for TNBC treatment. HDACs are known to inhibit the expression of DNA repair genes and tumor suppressor genes. Two clinical trials are investigating HDACi’s combined with DNA methyltransferase inhibitors and cisplatin [113]. Hsp90 has the potential to inhibit multiple growths, signalling and survival cascades. Phase 1 clinical trials with olaparib and paclitaxel for evaluating Hsp90 inhibitors are ongoing; however, Ganetespib (Hsp 90 inhibitor) reduced tumor volume in MDA-MB-231 derived xenografts [114]. Anti-androgens bicalutamide and enzalutamide, target the androgen receptor (AR) in various TNBC cell lines which further inhibited proliferation, invasion and migration of cancer cells suggested them as a surrogate biomarker for response to other therapies [115].Voltage-gated sodium channel (VGSC) isneonatal splice variant of the VGS subtype Nav1. 5. VGSC-inhibiting drugs such as ranolazine, riluzole and phenytoin, all suppress metastatic cell behaviours in vitro and/or in vivo and are the basis of clinical management of TNBC [116]. NP based formulations currently in clinical use for the treatment of metastatic breast cancer are Liposomes-Doxorubicin nanodrug as Lipo-Dox and Myocet was approved in 1998 (Taiwan) and 2000 (EMA) respectively for breast cancer [117]. In 2005-2008, nanoparticle albumin bound to Paclitaxel as Abraxane and doceaxel (DTX)-polymer NPs (BIND-014) [118] were approved for advanced metastatic breast cancer [119]. Similarly, using Paclitaxel as active drug, PEG-PLA polymeric micelle formulation as Genexol-PM was approved by South Korea in 2007 for breast and ovarian cancer. Numerous nanoparticles are being created for cancer treatment, and many of them are liposomal and polymeric nanoparticle platform. Liposomal nanoparticles with mitoxantrone named as plm60-s (Mitoxantrone HCl liposome injection) are in II phase of clinical trial for breast cancer [120]. LiPlaCisa liposome nanoparticle with cisplatin is in II pahse of clinical trial with the promising results for metastatic breast cancer [121].

### Immunotherapeutics: Targeting cancer with immune cells

Immunotherapy is a concept of utilizing intrinsic mechanism of host immune system to combat cancer by enhancing immune system to recognize and kill tumor cells. This is a novel and revolutionary discovery by 2018 Medicine Nobel Prize winners Professor Tasuku Honjo (Kyoto University of Japan) and Prof. James P Allison (University of Texas) for using immune checkpoint blockade to treat cancer by inhibition of negative immune regulation. Immunotherapy with recent advances has achieved success and yielded new therapeutics strategy for TNBC treatment [122]. Lack of targets for existing therapies and immunogenic nature of tumor, render them good candidate for immunotherapy. Various immunotherapies have been tested including immune checkpoint blockers, cytotoxic T lymphocytes (CTLs) activation, adaptive cell transfer-based therapy (ACT) and modulation of tumor microenvironment (TME). Such novel immune-modulatory strategies can tackle TNBC and emerged as personalized immunotherapy (Table 2).

**Table 2:** Ongoing clinical trials for TNBC: Different class of agents are being tested (eg. Anti-PD1- Pembrolizumab) on different subjects (eg. metastatic TNBC) and given in combination with other chemotherapeutic agents as the promising immunotherapy for TNBC treatment. These trials are approved with identifier and are in different phase of their evaluation with their probable compeltion date

Class of agents	Agent	Phase	Participants	Combinatorial agent	Completion	Identifier
AR antagonist	Enzalutamide	2	Stage I-III AR positive TNBC	-	April 2019	NCT02750358
Anti-PD1	Pembrolizumab	1/2	Recurrent TNBC, mTNBC	PLX3397 (TkI of KIR, CSFIR)	May 2019	NCT02452424
Anti-PD1	Pembrolizumab	2	mTNBC	Carboplatin + gemcitabine	June 2019	NCT02755272
Hsp90 inhibitors	Onalespib	1	Metastatic solid tumor, Recurrent TNBC	Olaparib	October 2019	NCT02898207
AR antagonist	Enzalutamide	1/2	AR positive mTNBC	Taselisib (PI3K inhibitor)	December 2019	NCT02457910
Anti-PD1	Pembrolizumab	3	Recurrent TNBC and mTNBC	Nab-paclitaxel /Paclitaxel/gemcitabine/ carboplatin	December 2019	NCT02819518
PARPi	Olaparib	3	TNBC, Germline BRCA1/2 mutation	-	March 2020	NCT02032823
PARPi	Olaparib	1b	Recurrent TnBc	AZD2014 (mTORC1/2 inhibitor)	November 2020	NCT02208375
AR antagonist	Enzalutamide	2	AR positive mTNBC	Paclitaxel	December 2020	NCT02689427
Anti-PD1	Pembrolizumab	2	Advanced TNBC or mTNBC	-	January 2021	NCT02644369
Anti-PD-L1	MPDL3280A	2	TNBC	Nab-paclitaxel	February 2021	NCT02530489
Anti-PD1	Pembrolizumab	1/2	mTNBC	Paclitaxel	May 2021	NCT02734290

#### Immunotherapy for triple negative breast cancer

Tumor vaccination comprises of tumor cells or tumor antigen, to stimulate host to produce effective anti-tumor immune response. This class of vaccine also includes DNA vaccines, anti-idiotypic Ab vaccine, anti-tumor related pathogen vaccine and dendritic cell vaccine. *Tumor cell vaccines* are produced from human tumor cells that retain its immunogenicity for better clinical outcome. Belanyenpumatucel-L is an example of allogenic tumor cell vaccine which specifically target TGF-beta-2 receptor [123]. *Tumor antigen vaccines* are developed by utilizing tumor specific antigen (TSA) and tumor associated antigen (TAA) [124]. A modified tumor antigen vaccine containing MHC1 is known as theratope is in phase III clinical trial. Endocrine therapy and theratope (Sialyl Tn-KLH; Biomira) combination showed slowing down of progression in metastatic breast cancer. *DNA vaccine* is a bacterium expressed plasmid with DNA encoding antigenic proteins which can elicit Ab or CMI response, for example Mammaglobin-A DNA vaccine in phase-I clinical trial for breast cancer treatment [125, 126]. Among the *anti-idiotypic Ab vaccine*, Racotumomab is against the surface membrane glycoprotein of NSCLC (Non-small cell lung carcinoma) and need to be explored for TNBC.

Apart from tumor vaccine, *T-cell based therapy* including adoptive cell transfer therapy (ACT) is the prime strategic response against cancer. ACT involves self-transfer of T cells, lymphokine-activated killer (LAK) cells, cytokine activated killer (TIL) cells, and macrophages activated killer (MAK) cells in patients to kill tumor cells and improve immune response. Cytokine-induced killer (CIK) cell infusion therapy with adjuvant radiotherapy had significantly prolonged disease-free survival in TNBC patients [127]. Modified approach of ACT is *cascade primed immune cell therapy* (CAPRI). As an adjuvant therapy, cells obtained from peripheral blood having tumor immunogenicity, become T cell and destroy the tumor cells of breast cancer [128]. Chimeric antigen receptor (CAR) can direct T cells to recognize antigen expression on tumor cells, however CAR therapy is associated with cytokine release syndrome (CRS), B cell aplasia and tumor lysis syndrome (TLS), thereby restricts the utility in cancer treatment [129].

*Cytokine therapy* treats cancer by multiple ways. The most common way is by elevating cytokines levels, enhancing expression of tumor-related Ag, by stimulating immune effector cells. IL-2 promotes T cell proliferation and activation of NK, CTL along with B cell proliferation [130]. However IFN-γ approved for treatment of renal cell carcinoma, CK therapy would be an attractive area in breast cancer treatment.

*Therapeutic antibodies* targeting CD3, CD19, CD20, CD22, CD30, CD33, epithelial cell adhesion molecule, VEGF, EGFR, HER2, NF-κβ, CTLA-4, PD-1 and PD-L1 receptors used as immunotherapeutics. Currently, herceptin and NeuVax vaccine (Immunodominant nanapeptide with GM-CSF) are targeted against HER2-expressing breast cancer. Zoledronate is in clinical phase–II for triple -negative breast cancer treatment [131]. Antibodies like cetuximab inhibiting EGFR; bevacizumab targeting VEGF; rituximab targeting CD20, and ipilimumab, nivolumab and pembrolizumab immunostimulating anti-CTLA-4, anti PD-1 and anti-PD-L1 respectively are the promising antibodies as immunotherapeutics for TNBC treatment.

*Immune checkpoints* are protective effector molecules of human immune system. Inhibitors of CTLA-4, programmed death 1 (PD-1) and programmed death ligand-1 (PD-L1) are immune check point blockers. Clinical trials with checkpoint inhibitors in breast cancer have only been recently initiated. CTLA-4/CD152 checkpoints are expressed on CD8 T cell, CD 4 T cell, Fox P3+ regulatory and NK cells involved in immune activation [132]. Ipilimumab (humanized IgG1MAb) and tremelimumab (human IgG2 MAb) are FDA approved antibodies used as CTLA-4 inhibitors in different cancer treatment which reactivate T cells and eventually enhance anti-tumor immune response. PD-1 is monomeric glycoprotein/checkpoint receptor, expressed by T cells surface and binding to PD-1 is blocked by blocker would enhanced T cell immune response [133]. Nivolumab (humanized IgG4 MAb) and pembrolizumab (humanized IgG4 isotype MAb) are FDA approved PD-1 targeting antibodies whichinhibit PD-1 and shows therapeutic benefit in melanoma and NSCLC in two different phase III trials (Checkmate-057 and Checkmate -037) [134]. Nivolumab is being evaluated as monotherapy in an adaptive phase II trial (NCT022499367) and in combination with TAK-659 (TK inhibitor) in phase 1b (NCT02834247) of metastatic TNBC [135]. Pembrolozumab blocks interaction between PD-1 and PD-L1/PD-L2 and evaluated as monotherapy by screening 111 metastatic TNBC patients in phase 1b (KEYNOTE-012; NCT01848834) trial [136]. Other trials assessing the efficacy and response of pembrolizumab as monotherapy in metastatic TNBC are phase II (KEYNOTE-086; NCT02447003) and phase III (KEYNOTE-119; NCT02555657) trials. Combination of pembrolizumab and chemotherapy is also evaluated in randomized phase III (KEYNOTE-355; NCT02819518) trial for metastatic TNBC. Effect of adjuvant treatment with pembrolozumab is also evaluated in phase III (SWOG-S1418, BR006; NCT02954874) trial with 1000 TNBC patients. Higher levels of tumor-infiltrating lymphocytes (TILs) have prognostic significance and suggest immune response to tumor associated antigen in TNBC [137]. PD-L1 expressed on tumor cells exerts inhibitory effect on T cell and tumor-infiltrating inflammatory cells by interacting with PD-1 receptor on T cells. Clinically important PD-L1 inhibitors are atezolizumab (IgG1 isotype MAb), avelumab (human IgG1 MAb), and durvalumab (IgG1 MAb). Atezolizumab binds selectively to PD-L1 on immune cells/tumor cells and prevent interactions with the PD-1 receptor. A phase I (NCT01375842) trial with fifty-four metastatic TNBC patients [106] to assess the safety profile of atezolizumab and phase 1b (NCT01633970) trial in combination with nab-paclitaxel emerged as attractive chemoimmunotherapy in metastatic TNBC treatment [138]. Combined efficacy of atezolizumab and chemotherapy is evaluated for TNBC in phase III (NCT02620280) neoadjuvant trial. Recently, FDA granted approval to first immunotherapy i.e. Atezolizumab (Tecentriq, Genetech/Roche) plus chemotherapy nab-paclitaxel (Abraxane, Celgen) for the first-line treatment of unrespectable locally advanced or metastatic, PD-L1-positivetriple-negative breast cancer (TNBC) [18]. Atezolizumab plus nanoparticle albumin-bound (nab)-paclitaxel synergistically enhance the anticancer activity and prolonged the progression-free survival among patients with metastatic triple-negative breast cancer in both the intention-to-treat population and the PD-L1-positive subgroup (Impassion130; NCT02425891). Velumab is undergoing a phase Ib (JAVELIN; NCT01772004) trial in a cohort of 168 metastatic breast cancer patients. Another phase III randomized trial (A-BRAVE; NCT02926196) was conducted in 355 TNBC patients with avelumab to evaluate adjuvant treatment. Durvalumab blocks the activation of PD-1 receptor expressed on activated T cells. Various clinical adjuvant therapeutic trials are going with different stage TNBC patients. Phase Ib (NCT02826434) trial for stage II/III TNBC patients include durvalumab with PVX-410 vaccine as adjuvant; however, another phase I/III (NCT02489448) trial with neoadjuvant nab-paclitaxel with doxorubicin, cyclophosphamide and durvalumab in stage I/III TNBC patients. Other targets like lymphocyte activating gene 3 (LAG3) and T cell immunoglobin and mucin-3 (TIM-3) are expressed on activating T cells, NK and monocytes and served for immune checkpoint inhibition [139].

Immuno-interventions are being explored as neoadjuvant therapy against TNBC. Melanoma-associated antigen-3 (MAGE-3) and alpha-lactalbumin antigen are expressed in breast and tested as a tumor vaccine to produce effective anti-tumor immunity. Significant suppression of breast tumor is reported in mice vaccinated with GM-CSF adjuvant alpha lactalbumin vaccine [140]. Allogenic Dendritic cell (DC) fused TNBC vaccine can stimulate T cell proliferation and produce tumor specific immune response against TNBC, possibly by increasing IL-12 aand IFN-γ levels [141]. Many clinical trials of immunotherapy agents are in progress with a hope to change the standard of TNBC care and treatment.

#### Nanomaterials assisted immunotherapy for TNBC

Nanotechnology provides efficient and smart nano-delivery systems facilitating the delivery of immunostimulating adjuvants and tumor antigens to enhance antigen presentation and immunity which aids in treatment of metastasis. An improved and clear understanding of TNBC immunogenicity has led several trials with different immunotherapeutic agents, with the hope of developing new immunotherapeutic modalities in TNBC [137, 142]. Approximately, 45 formulations, majority of liposomal NPs containing GM-CSF, anti-TNF-α are approved for clinical use in cancer therapy. Nano-particulate carriers improve solubility and bioavailability of immunotherapeutic and protect them from degradation, therefore enhance potential efficacy.

Nanoparticles (NPs) assist improvement in antigen expression pathways by the delivery of epigenetic modulators and immunostimulator cytokines [108]. NPs mediated transfer of epigenetic inhibitors has been efficacious in initial trials of breast cancer. DOX with decitabine NP (DNMTi) shown to increase the sensitivity of breast cancer cells [143]. Similarly, vorinostat (HDACi) delivered with improved solubility (four-fold), half-life and pharmacokinetics using the poly-ethylene-oxide-polylactic acid (PEO-PLA) copolymer micelles [144]. Cytokines like IL-2, IFN-γ,TNF-α and thymosin are FDA approved immunostimulator for cancer treatment (renal cell carcinoma). These cytokines directly stimulate NK, CTL and immune effector cells and finally enhance the immune response. Liposomal NPs mediated delivery of cytokines, for example PEG-coated liposomal NPs assisted delivery of IL-2 cytokines has reduced tumor growth [145]. Cytokine therapy in combination with cancer vaccines may able to stimulate and increase effector T cells, but still more research needs to be done for TNBC nanomedicine.

Tumor micro-environment (TME) is the critical factor which affects the delivery and efficacy of diagnostic and therapeutic modules. NP mediated delivery of soluble mediators like TGF-β receptor inhibitor broadly increases the number of CD8+ T and NK cells. Other strategy to knockdown TGF-β by 50% in melanoma using liposome-protamine-hyaluronic (LPH) acid NP co-delivered with siRNA (TGF-β) and cancer vaccine [146]. Stimulator of IFN gene (STING) resides inside the cell and is becoming an exciting target for cancer therapeutics. cGAMP encapsulated liposomal NPs (cGAMP-NP) penetrates inside the cell for its intracellular delivery. cGAMP-NP directed activation of STING, activate human macrophages to increase IFN-γ producing T Cells which eventually reduce melanoma tumor load [147]. A self-degradable hyaluronic acid (HA) integrated pH sensitive dextran NP patch which encapsulates PD1 and glucose oxidase (GOx) was developed as immunotherapeutic module by Wang et al.,[148]. In melanoma mouse model (B16F), a strong robust immune response was induced with this novel microneedle patch. CTLs response in cancer therapy is also induce by lipid-calcium-phosphate (LCP) NPs mediated transfer of cancer antigen. B16F10 melanoma also treated with lipid NP formulation containing mRNA for gp100 and TRP2, thereby inducing strong cytotoxic CD 8 T cell response resulting in overall shrinkage of tumor in mice [149].

*Inherited immunostimulating immunotherapeutic* involve metals like selenium (Se) and mica posing inherit immunostimulating properties. SeNPs exhibit its anti-cancerous activity by stimulating neutrophils, T and B lymphocytes and NK cell mediated cytotoxicity [150]. However, the oral and nasal mode of administration is the major drawback of this therapy and needs re-evaluation in cancer immunotherapy.

Other nanocarriers facilitating the immunotherapy are liposomes, exosomes and nanospheres. Cationic liposomal NPs with poly (I/C) and peptide emerged as cancer vaccine formulation showing increased T-cell response [151]. Fc receptor targeting tumor peptide vaccine (nano-liposome) with Palm-IL-1/MAP-IFN-γ peptide as adjuvant, targets the DCs and produce strong anti-tumor response in cancer patient. Ascetic cell exosomes (small membrane vesicles) also induce production of cytotoxic T-lymphocytes when given with granulocyte-macrophages colony-stimulating factor (GMC-SF) in phase 1 clinical trials [152]. Combined delivery of siMDR1 (multi-drug resistance gene) with DOX using hollow carbon nanospheres facilitates 90% reduction of tumor weight in mice by down-regulating MDR1 protein expression [153]. Even the antigen-capturing nanoparticles (AC-NPs) induce CD8+ cytotoxic and CD4+ T cells population, thereby improving cancer immunotherapy [154].NP carrying bevacizumab and CRLX101 showed good efficacy in TNBC treatment [155]. Polymeric NP based vaccine with IFN-stimulated gene and albumin NPs with TA99 Mab can turn phagocytes and lifting neutrophils against cancer [156, 157].

*Autophagy* is known to promote or suppress cancer development (double edge sword), therefore recently explored as immunotherapeutic modality. Autophagy is a genetically well controlled defence mechanism which has been reported to modulate immune system. Sulforaphane (SFN) induce autophagy by down regulating the expression of histone deactylase (HDAC6) mediated phosphatase and tensin homolog (PTEN) activation in MDA-MB-231 and MDA-MB-468 cells which significantly sensitizes TNBC to DOX. Autophagy induction (SFN) in combination with DOX (therapeutic) inhibits tumor growth and may provide an effective approach for TNBC therapy [158]. Autophagy mediated suppression of cancer is promising treatment modality and warrants detailed investigation.

### Nanoparticle infiltration: Route from blood vessels to breast tumor site

Nanoparticles (NPs) with ligands for targeted drug delivery or carrying diagnostic and/or therapeutic (theranostics), or loaded with immunotherapeutic with immunomodulating or immunostimulating anti-cancer affects needs to be injected, circulated in blood vessels and finally needs to be targeted at the cancer site by crossing or travelling the endothelial barrier. Size, shape, charge and density of nanoparticles are the important parameters which decide the trajectory, dynamic, stability and distribution while circulation in blood stream and subsequent mechanism of infiltration in tumor tissues and cells. To reach the tumor site and to successfully deliver the therapeutic drug, vascular barrier needs to be crossed.

NPs usually follow two different strategies i.e. passive route and active route to target cancer site. Majority of the nanomedicine assumes and follow the passive phenomenon known as enhanced permeability and retention (EPR) route which is totally dependent on the tumor type and stage of cancer. In EPR effect, NPs passively cross leaky vessels of tumor tissues and accumulate into the cancer cells. Degree of leakiness depends on the tumor location, vasculature and progression stage [159]. Nano-carriers once injected for the systemic delivery of cancer therapeutics; in the circulation they are coated with plasma proteins and readily available to clearance by mononuclear phagocyte system (MPS). NPs avoid MPS by PEG surface coating and this NP extraverted from tumor blood vessels into tumor by margination (ability to flow towards blood vessels walls) leading to longer half-life through prolonged circulation in blood [160]. Inside tumor cell, NPs release their cargo by the process of particle erosion and diffusion. Non-spherical particles (100 nm) marginate more rapidly and extravert through the leaky tumor vasculature and penetrate into tumor mass. Surface modification involving zwitterionic ligands eg. cysteine and glutathione or PEGylation facilitate escape of NPs from reticuloendothelial system (RES) and finally reach the target tumor tissue [161]. Even poor lymphatic drainage system provides permeability to cross the barrier and allow NP to passively diffuse/penetrate to the target the cancer site. So, EPR effect is mostly seen in mature tumor and attributed by the nutrient-starved condition in tumor.

**Fig. 4:**
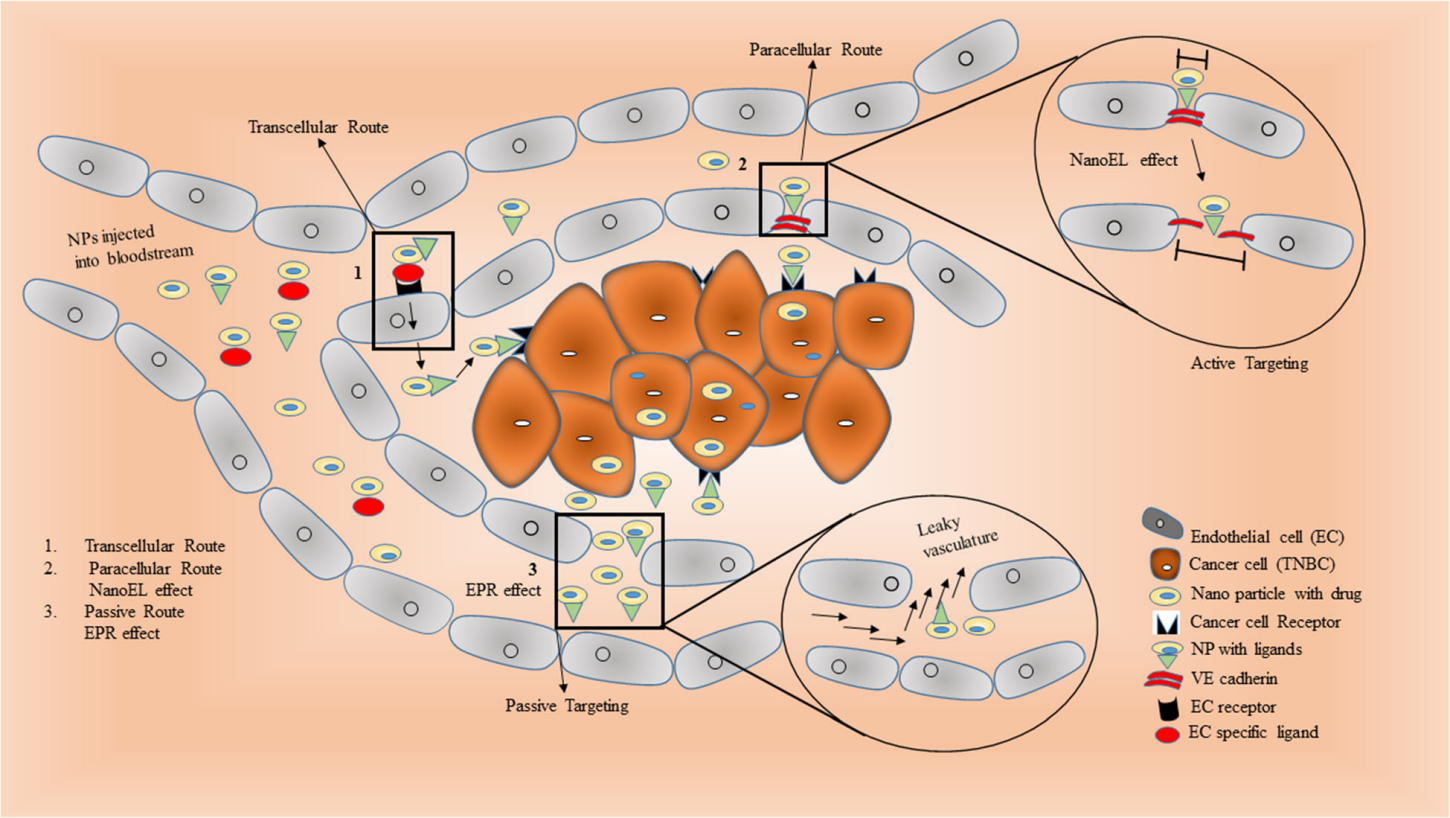
Schematic representation of different routes followed by nanoparticles in cancer therapy. (1) In transcellular route, the drug carrying nanoparticle injected into the bloodstream passes through the endothelial cell and reaches as the cancer site. (2) Whereas in paracellular route, the nanoparticle passes through the inter-endothelial cell spaces and known as active targeting. These nanoparticles induce leakiness by widening the gap between the endothelial cells and enhance the cancer cell targeting also known as naoEL effect. (3) Passive targeting is the classical and common phenomenon where the nanoparticle transverse through the leaky vasculature by EPR effect to the site of cancer cells.

Contrary to above passive route, greater selectivity and specificity for cancer cells is achieved surface modification of NPs with ligands like transferrin, folic acid and antibodies for specific targeted therapy on glycan surface of the tumor cells [162]. Transcellular route and movement between the endothelial cells are the newer strategies. Receptor mediated internalization is facilitated by the endothelial cells (ECs) surface receptors following transcellular transport across the EC barrier is accompanied with certain shortcoming like lysosomal digestion in EC cellular processing and few EC specific markers. Therefore, more profound mode is paracellular route having VE-cadherins and occludins junctions across the narrow intercellular spaces between two endothelial cells (EC). This gap between the ECs is the new target and needs to be widening by NP to access the tumor site. So, for, targeting early and benign cancer, nanomaterial induced endothelial leakiness (NanoEL) phenomenon is emerging. NPs are now designed to induce endothelial leakiness forming capillary beds without EPR effect. Physiochemical intrinsic properties of NPs like charge and density regulates the NanoEL and cancer progression. Gold NPs (10-30 nm) is recently exploited as NanoEL inducing particles in human mammary endothelial cells [163]. Using charge as an important entity, AuNPs charge (negatively and positively charged NPs) could be tuned for NanoEL effect. Negatively charged gold NPs (-AuNPs) could be attracted and bound towards positively charged cell-cell junction and could induce NP driven leaky effect to access the tumor. Using the same concept, positively charged AuNPs (+AuNPs) are attracted by glycocalyx and modulate the degree of NanoEL effect by endocytosis (paracellular route) into the endothelial cells (EC) barrier. However, based on bouncing particle hypothesis negatively charged NP (-AuNPs) caused more NanoEL effect (Fig. 4, [164]).

Micron sized gaps between endothelial cells could be easily passed by NPs but not the nanosized gaps between microvascular capillary. Therefore, size of nanoparticle is another important feature, as smaller sized NPs can easily penetrate and accumulate in the leaky tumor vessels. Even density of the NPs dictates the NanoEL effect. Using SiNPs library of varying densities, *Tay et al*, [165] reported high endothelial permeability with particle density between 1.57 g/cm3 to 1.72 g/cm3. Same group of researchers tried various small sized (15-25nm) NPs like titanium dioxide (TiO_2_), silica dioxide (SiO_2_) and silver (Ag) for NanoEL effect. These NPs randomly entered nanometer wide gaps of the adherens junctions between endothelial cells disrupt the VE-Cad-VE-Cad interactions and produce micron sized gaps between endothelial cells. *Setyawati et al.*, [166] also demonstrated a novel non-receptor mediated endothelial cell leakiness (ECL) by TiO_2_ NPs targeting specific interaction with VE-cadherin protein. TiO_2_ NPs migrate into the inter-endothelial adherents junction niche and binds directly to VE–cadherin resulting in cascade of intracellular reactions and finally disruptcell–cell interactions. This facilitates application of the nanomedicine for the treatment of cancer. Surface re-modelling of the NPs like addition of chemical groups on the surface of Nanodiamonds also known to modulate the degree of leakiness in the vascular barrier. Such modified nanodiamonds widen the paracellular route opening of EC barrier by increasing the intracellular Ca^2+^and ROS, inducing the cytoskeleton remodelling resulting in vasculature leakiness [167]. This demonstrated the possibility for doxorubicin to penetrate effectively through leaked vascular barrier to reach the cancer cells with high efficacy of drug delivery.

NanoEL effect although is beneficial in enhancing the delivery of drug carrying NP, but it’s no specificity to induce spontaneous leakiness in other blood vessels could have adverse effects. Studies by *Setyawati et al.,*[163] also showed side-effects of TiO_2-_NanoEL induced leakiness of subcutaneous blood vessels in mice and enhanced circulating melanoma metastasis to lungs in the mouse model. These inherited side-effects of NPs induced non-specific NanoEL effect and high probability of interaction with biological tissues has raised safety concerns which need to be addressed. Designing of specific and smart NanoEL driven nanoparticles can be the future of cancer nanomedicine which could target the wide spectrum of tumor including TNBC.

### Novel research for cancer detection and treatment: In news

Scientists from University of Queensland developed a method to detect cancer in 10 min using blood sample with accuracy of 90%. They developed such sensitive detection test using simple colour changing fluid to reveal the presence of malignant cells based on the hypothesis that normal DNA and Cancer DNA shows different adherence to metal surfaces and stick differently [168]. In addition, researcher from Rosalind Institute developed a revolutionary high-speed camera that can take 100 million photos per sec at 1-megapixel resolution across the spectrum from Infra-red (IF) to ultra-violet (UV). This high-speed camera helps the researchers to see how a drug reacts with a cancer tumor at a microscopic level in Real-Time [169]. Recently, Israeli scientists claim to develop 100% cancer cure by early 2020, using a treatment known as MuTaTo which stands for Multi-Target-Toxin [170]**.** This treatment is developed by Accelerated Evolution Biotechnologies Ltd (AEBi) based on the SoAP Technology. Successful preliminary trials on mice using a combination of cancer targeting peptide and MuTaTo toxin, showing the specificity towards cancer cells and terminate cancer cells without harming normal healthy cells and tissues. The concept that makes MuTaTo treatment different from existing treatments is the attacking cancer cells receptors from 3 different directions simultaneously at the same time and reflect the scope of hyper-personalized treatment to each patient in the long run. Recently the researchers from the University of Queensland and Albert Einstein College of Medicine jointly developed a statistical approach known as Oncomix [171] to examine breast cancer data from The Cancer Genome Atlas patient database and identified the most promising target known as Chromobox 2 (CBX2) which has been shown to have high levels in aggressive sub-type of breast cancerand therefore could be a potential breast cancer treatment target**.** Oncomix, captures transcriptional heterogeneity in tumor and identifies oncogene candidates that were over expressed in a subset of breast tumors. Intronic DNA methylation was strongly associated with the over expression of Chromobox 2 (CBX2), an oncogene. CBX2 over expression in breast tumors was associated with the up regulation of genes involved in cell cycle progression and with poor 5-year survival [171]. This discovery highlights the potential value of the Oncomix approach and will open new therapeutic avenues and move us closer to personalized medicine.

#### Artificial intelligence (AI): Advanced cancer diagnosis and treatment (Futuristic approach)

**Fig. 5:**
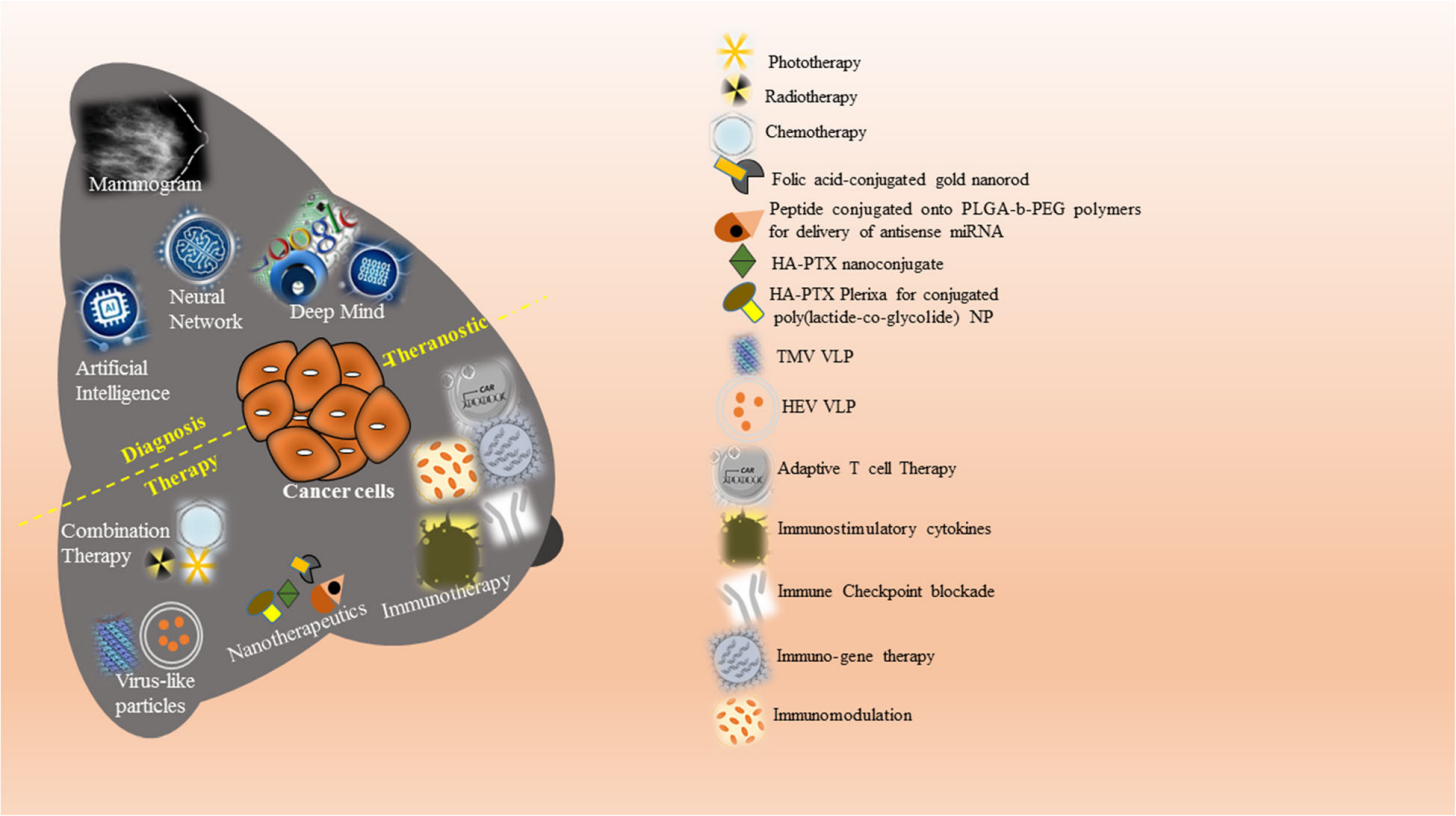
Graphical Abstract: Advancements in the theranostics: Recent advancements like artificial intelligence, neural network and deep mind in addition to classical mammogram predict and improve the breast cancer diagnosis. Additionally, immunotherapies using immune checkpoint blockade, immunostimulatory cytokines, and adaptive cell therapy in addition to current combination therapy collectively improve the diagnosis and treatment when articulated in the form of theranostics.

Artificial intelligence revolutionizes every bit of science whether it is engineering, robots, defence, nanotechnology or medical science. Everything is now going smart whether it is our phones or watches, thanks to artificial intelligence. Recently, Cancer Research UK Imperial centre, DeepMind Health, AI health research team at Google and UK funded OPTIMAM mammography database at the Royal surrey country hospital NHS foundation trust collaborated to improve breast cancer diagnosis using AI [172]. Machine learning technology from DeepMind Health and de-identified mammograms, possibly train computer algorithm to analyse these images more accurately, leading to earlier detection and therapeutic intervention for patients (Fig. 5). However, AI in medical imaging is still in its infancy, but such collaborations will soon develop cutting-edge machine learning to detect and diagnose breast cancer more selectively and accurately. Scientists at Imperial College London and the University of Melbourne developed machine learning software known as Radiomics Prognostic Vector (RPV) that can predict the prognosis of 364 ovarian cancer patients four times as accurate at predicting outcomes when compared to conventional methods in an initial trial by examining four biological features of tumor including structure, shape, size and genetic makeup in CT scans [173].

#### AI guided nano-robots for cancer treatment: Hypothetical view

Artificial intelligence (AI) guided nano-robot or self-learned/evolved, made of biocompatible/ degradable material (carragennin/capsule coat) which can carry drug specifically to the target cancer site must have all ancillary like sensor for the target, tracking sensor, self-detonation property to get rid of from the body after serving the required purpose. In near future, nano-medicine research will be powered by AI, not only to diagnose and treat cancer, but to deal with all other disease. Even though much success is achieved with the nanotechnology in cancer research and treatment, the intervention of artificial intelligence in nanotechnology could be the promising solution.

## Conclusions

Triple-negative breast cancer still consider as an aggressive subtype of breast cancer. The high heterogeneous nature accompanied with low survival rate continues as a challenge to the oncologist. Currently available therapies are inadequate and needs to be supplemented with novel targeted therapies to tackle the tenacious TNBC tumor. With the emergence of nanoscience, nanomedicine is likewise advancing in terms of accurate and rapid diagnosis and target directed remedy in cancers.

Nanoparticles are the key players in most cancers research due to its target specific multifunctional properties. These nano-missiles are well equipped with arsenals to execute their role in destroying the most cancer cells. The possibility to load/encapsulate drug, not only protect drug but additionally increase biological half-life of anti-cancerous drug which eventually lower the overall dose of drug administration. Such encapsulation aids the slow and concentrated release of drug at cancer site due to enhanced permeability and retention (EPR) thereby reducing the side-effects to other non-cancerous healthy cells. The selected targeted delivery of drug increases treatment efficacy. Versatility in terms of size, materials used, fabrication technologies, in addition to biocompatibility and biodegradability certified these nanocarriers for cancer diagnostics and therapeutics. Successful designing of dual-functionality nanoparticles for simultaneously monitoring (imaging) and treating (drug) of cancer: theranostic had been developed with a very promising future in cancer. High multiplexibility by conjugating ligands to nanoparticles facilitates the combined targeted delivery of drug at precise site to selectively destroy tumor cells.

Demonstrating the diverse application, still there are few challenges which are needing to be addressed. Majority of the nanovehicles in TNBC trials are designed either for targeted diagnosis or targeted therapy. A very few studies demonstrating the utility of these nano-vehicles are conducted in *in-vitro* TNBC cell line and in *in-vivo* xenograft mouse models. Limited TNBC (cell or animal) model simulating the actual clinical situation is still a challenge and to be addressed promising immunotherapy with the recently approved drug and in-trials drugs surely will limit the cancer progression and advance the treatment. Even the better understanding of how the nanoparticles enhance and mediate the immune response also improvises the TNBC treatment. Expertise in integrating various modalities in one system and understanding the molecular and cellular interaction is still a limitation which needs a promising solution. Success of few drugs for breast cancer also showing promising results with TNBC cell culture models, these endeavours successfully motivates the concept of drug-repurposing for TNBC treatment. Finally, nanotechnology-based drug delivery with enough ancillary (drugs, ligand, and probe) system can improve diagnostic ability and therapeutic outcomes, thereby contributing to enhanced patient survival and well-being.

## Data Availability

Not applicable

## Abbreviations

ACT: Adaptive cell transfer-based therapy
AI: Artificial Intelligence
AODNs: Antisenseoligodeoxynucleotides
BC: Breast cancer
CAR: Chimeric antigen receptor
CIK: Cytokine-induced killer
CTLs: Cytotoxic T lymphocytes
EPR: Enhanced permeation and retention
ER: Estrogen receptor
FDA: Food and Drug Administration
GFP: Green fluorescent protein
HCC: Human Hepatocellular carcinoma
HDAC: Histone deacetylase
HER-2: Human epidermal growth factor receptor-2
IHC: Immunohistochemistry
LAG3: Lymphocyte activating gene
LAK: Lymphokine-activated killer cells
MAGE: Melanoma associated antigen-3
MAK: Macrophages activated killer
MPS: Mononuclear phagocyte system
MRI: Magnetic resonance imaging
NanoEL: Nanoparticles induced endothelial leakiness
NIR: Near Infra-red
NSCLC: Non-small cell lung carcinoma
o-MWNTs: Oxidized multi-walled carbon nanotubes
PARP: Poly (ADP-ribose) polymerase
PD-1: Programmed death 1
PD-L1: Programmed death ligand-1
PEG: Polyethylene glycol
pHLIP: pH low insertion peptide
PR: Progesterone receptor
PRINT: Particle replication in nonwetting templates
PTX: Paclitaxel
RES: Reticuloendothelial system
ROS: Reactive oxygen species
RT: Radiation treatment
RTK: Receptor tyrosine kinase
TAA: Tumor associated antigen
TCM: Terahertz (THz) chemical microscopy
TK: Tyrosine kinase
TLS: Tumor lysis syndrome
TME: Tumor microenvironment
TMT: Tumor metastasis targeting
TNBC: Triple-negative breast cancer
TSA: Tumor specific antigen
uPAR: Urokinase plasminogen activator receptor
VCAM-1: Vascular cell adhesion molecule-1
VEGF: Vascular endothelial growth factor
VLP: Virus like particles
